# Dr. Conolly on the Indications of Insanity

**Published:** 1830-10-01

**Authors:** 


### Medico-Chirurgical Review,

### No. XXVI.

JULY 1 to OCTOBER 1, 1830.

### I.

An Inquiry concerning the Indications of Insanity, with Suggestions for the better Protection and Care of the Insane. By *John Conolly*, M. D. Professor of Medicine in the University of London. Pp. 495, 1830.

"Hic homines ex stultis facit insanos."

Soundness of mind is a comparative phrase. What appears sensible and rational to one mind appears neither rational nor sensible to another, and we deem it to be a task of as great difficulty to fix the standard of judgment as of taste. The eccentricity which we easily detect in another, may long remain unnoticed in ourselves; and opinions, which we ardently cherish and zealously support, others may regard as hastily formed or totally unfounded. The man, who believed himself to be a crown-piece, and went round his tradesmen, requesting them not to accept it if offered in payment, would nevertheless ridicule his neighbour, who fancied that his legs were made of butter, or his nose of sealing-wax. Few men are unexceptionably sane, just as few men are unexceptionably healthy. Some predominance of one faculty or passion, some superiority of one function or organ above another, may be discovered, and we can see no very good reason why an insignificant coxcomb, who has an immoderate opinion of his merits, should not have the same care taken of him, as a beggar, who fancies himself a duke or prince; or why a man, who starves and languishes in the midst of plenty, should be trusted to his own keeping more than he who imagines himself an emperor in the midst of poverty.

This important fact seems almost entirely to have been neglected, and most of those, who have written upon insanity, seem to consider it as a well-defined and specific malady, so palpably pronounced and so prominently featured, that few should find much difficulty in discovering it when present, or easily mistake it when once observed. But the truth is that there are just as many grades of mental derangement as there are of mental perfection. One mind excels in imagination, another in memory;—in one it is the judgment which is weak, in a fourth it is perception. It is not required of us to state wherein these differences consist, or whence they originate:—whether they arise from original modifications in the metaphysical construction of man, or depend upon the various degrees of perfection in which his corporeal organization exists. May not mind, which is as much a creature as matter, although endowed with a different nature and subject to different laws, be given in different degrees of power, if not in different quantities; and may not the metaphysical part of a human being be primarily subject to as many and as great varieties of original construction as his physical part? We see people, at first educated and afterwards living as exactly under the same circumstances as it is possible to arrange them, differing as much from each other in talent, taste and habit, as does the number of their pulse, or the color of their hair; and is it not most rational to account for those mental varieties, just as we explain these corporeal differences—upon original modifications of structure and function? By the mere Materialist, who believes that all the metaphysics of man are the result of his bodily mechanism, and by the semi-Materialist, who more liberally admits the existence of an independent spirit, but most strangely maintains that mind—the thinking, reasoning, and only actively spiritual part of man—has no connexion with the soul, or this independent spirit, but is a mere function of the brain—this view of the question can scarcely be opposed. For if mind be the function of the brain, and if ideas be the product of this function, it is evident that both the product and the function will depend for their energy and extent on the activity and health of the cerebral organs. But the cerebral organs will be admitted to be subject to and to present all the varieties of strength, activity, and soundness which are discoverable in any other form of glandular structure; and it is unnecessary to add how endless and how great these varieties are! Consequently, the doctrine—that mind is originally the same in all men—cannot be maintained by any, who regard mind as the sole result of mechanism; and we can see no unanswerable argument in its favor to be advanced by those, who advocate the spirituality as well as the materiality of man. In either case, however, the fact remains unaffected, that the talents and tastes and habits of different men do differ from some cause, and it is equally certain that these varieties are as universal in their occurrence, as great in their degree, and as important in their operation as are those corporeal varieties which distinguish nations and individuals. To constitute, or preserve a healthy state of body every organ must be firmly braced, and every function must be finely balanced; and to constitute, or preserve a sane state of mind the brain, or organ of mental manifestation, must not only be well formed and in good health, but every faculty of the mind must bear a natural proportion to each other. It is the want of this proportion which constitutes eccentricity, and the limits between eccentricity and madness are very imperfectly marked.

Nimium insanus paucis videatur, eo quod,
Maxima pars hominum morbo jactatur eodem,—

are the words of Horace, than whom few were more accurately acquainted with human nature; and it matters not whether mental disorder be denominated simplicity, or eccentricity, or idiotcy, or monomaniasm, or madness, it is essentially the same. It may assume the form of some trifling peculiarity, which may be seldom observed as it may only occasionally appear; or it may wear the character of some ludicrous hallucination, which taints and tarnishes every action and thought. It may be limited to a single faculty, or it may invade every element of the mind; it may be elicited by certain subjects and circumstances, or it may so engross the character, as to be at no period absent or inactive.

One of the chief objects which Dr. Conolly has contemplated in the work before us is to show, how intimately sanity, eccentricity, idiotcy and madness are connected, and how easily they pass into each other. In endeavouring to effect this he goes into a short analysis of mind, and traces up its history through all its aberrations, whether congenital or induced. In the child every faculty of mind, as well as every corporeal function is imperfectly developed. At first the infant displays very little intellectuality, beyond what is connected with *sensation*, and *perception* which is the result or effect of sensation upon the brain. Then follows *memory*, or the power of preserving and recollecting these perceptions; afterwards appears the faculty of classing, arranging and multiplying these perceptions, which metaphysicians denominate *association*; and last of all is added *judgment*, which discovers resemblances or differences between these perceptions, and draws inferences accordingly. All these faculties thus successively developed gradually increase. Sensation becomes more exact from experience, perception gains acuteness by exercise, memory expands as sensations multiply, and the judgment, which is the last and slowest in coming to maturity, acquires strength by the frequent formation of comparisons.

The power of these faculties varies exceedingly, and their natural varieties are rendered more conspicuous by many accidental causes. The experienced seaman can easily distinguish ships, between which the landsman is unable to detect any points of difference. The Peruvian can discover by his sense of smell the race, or country of many South American tribes. The Arab can observe the caravan approaching in the desert, before it is visible to another eye. The blind can often detect colors by the sense of touch. The man described in the Spectator, who could not dance except in the presence of a trunk, which had formed part of the furniture of the apartment in which he had been taught, is one of many illustrations which might be given of the influence of habit upon mind. Sacchini was never inspired, but when his favorite cats were reposing on his shoulders: and Rush mentions the cases of a clergyman and a judge, who were always mad, except when in the pulpit, or on the bench: Brindley, the engineer, betook himself to bed for a few days when any extraordinary exercise of mind was necessary; Ben Jonson wrote with most effect after recovering from a debauch; Sheridan was most eloquent when under the influence of wine; Curran spoke with greatest power after playing on the violoncello; and Gluck composed his sweetest harmony in a meadow, with some champagne at his elbow and a piano before him.

There is another primary faculty of mind which has not yet been mentioned—we mean *imagination*. This heaven-born power is purely and unmixed the gift of Nature. No tutoring of the mind can unfold it; no course of study, no labor of thought, no stores of knowledge can create it. To be enjoyed it must be given; but when given it may be improved. Since the exercise of this faculty has the most intimate connexion with the subject of insanity, and as the following passage owes to it much of its beauty, we cannot refrain from extracting the Professor's vivid portrait of the "Poet's Gift."

"Under proper regulation, it is to its possessor a gift of exceeding and incalculable value; so augmenting every external sense as to be like the addition of one more than is possessed by his fellow-creatures; and giving to his recollected sensations and feelings a vividness of which lower organizations are quite incapable. It imparts energy to every faculty, and, strictly guarded by their action, ministers to them even in all the business of life: it inspires the thoughts of the poet; it dictates to the orator; it breathes on the ear of the composer harmonies never heard on earth before; it directs the sculptor to the beauty which lies buried in the shapeless block, unseen by any eye but his; it cheers and supports through excitement, through anxiety, in watchings and labours impossible but for the help which flows from this eternal source; and it leads each in his own language to the expression of that ideal beauty which fills his mind, and which none else can feel or represent. In the absence of all other revelations of the Creator's will, this faculty it is which leads man, in sage or simple or savage state, to some conception, some adumbration and foreknowledge, of a state beyond this life, where what can only be felt here, may be a blest reality. Whilst to the man who possesses no imagination, a flower is a mere flower, and night and day are but a succession of light and darkness, and the landscape is but so many acres of various coloured earth; he knows only that the flowers appear because it is the season of spring, and the leaves fall because it is autumn, and the storm rages because it is winter. But the man of glowing imagination is penetrated with all the undefinable influences of external nature,—of the morning and evening, and the deep night, and of every changing season; and associates all these with those images of greatness and that unattainable moral beauty which, all inconsistent and wayward as he may be, for ever exist within his soul. He finds, in the simplest flower that blows, a volume of contemplation: the scattered leaves present him with lessons of mortality: he hears the voice of God in the wind. He penetrates to the mysterious meanings of all that meets the mortal sense, and has sympathies of thought which never yet were uttered in words. Without losing his consciousness, as men in sleep, he can exercise the boundless power of fancy in his study, or in his walks, or in the crowd; create imaginary characters, invest them with life, animate them with feelings, inform them with eloquence; or, exhausting all the materials of this world, he can wander into regions to all else forbidden: the portals of hell admit him to the dreadful secrets within; or he travels in the immeasurable spaces between the everlasting stars, and the gates of heaven turn on their 'golden hinges' to receive him." 82.

All the mental faculties may thus be most variously modified, and the result of this diversity will be that, without any mental disease, men of every variety of talent may be met with. In no such case has the healthy balance been destroyed, and therefore in no such case can the mind be considered insane, although it may be weak. Perception discerns with accuracy, memory recollects with faithfulness, judgment draws sensible comparisons upon all ordinary subjects, and imagination operates under the control of reason. A man may be born, or he may become blind or deaf, and must, therefore, be rendered incapable of drawing comparisons and conclusions between sounds or colors; but so long as the man remains sensible of his defect, and declines expressing his opinion on points with which he knows himself to be unacquainted, he is sane in mind, although impaired in sense. A lady could not endure the touch of velvet. A Russian general could not suffer the sight of mirrors. Dr. Reid had a patient, who was so averse to every thing of a light color, that he begged the Dr. to muffle his white stockings in a black apron before coming to visit him. These peculiarities depended on some defect in the organs of sensation. The apparitions of Nicolai of Berlin, and the false perceptions of Sir J. Reynolds, after being for a long time confined to his pencil, are attributable to deranged perception. A striking instance of the loss of memory occurs in the late John Hunter.

For nearly half an hour he could not recollect the name, or situation of the street in which he was, nor could he even recognise it when mentioned to him. The patient mentioned by Dr. Reid, who spent all day in his bed because he could not decide upon the pair of trowsers he should put on, was a man of weak judgment. The man described by Dr. Haslam, who could not engage his attention for any time on a single point, but ran without connexion over every subject which his fancy suggested, was labouring under a defect in the faculty of association. And Ben Jonson, who kept awake for an entire night gazing at his great toe, round which he fancied he saw Turks and Tartars, Romans and Carthaginians contending, was a man of inordinate imagination. In all these instances the judgment is so triflingly and so transiently disordered, that its derangement does not amount to insanity. The transition, however, between this state of mind and that of insanity is easy and almost imperceptible. When a man can occupy an entire night in amusing himself with a visionary diorama of different nations engaged in battle around his toe; or when he can consume an entire day, before he determines on the pair of trowsers he is to wear, his judgment is unquestionably on the verge of ruin, and differs but little from that of the man described in the Spectator, who committed suicide to escape the daily misery of tying his garters; or of the self-complacent lunatic, who believes himself a king issuing his commands to all around him with the voice of majesty.

Insanity is one of those affections which are more easily described than defined. Perhaps it may not be asserting too much to say that every attempt, which has been hitherto made to condense its distinguishing characters within the limits of a definition which can comprehend every instance of derangement, has proved unsuccessful; and when the difficulties in the way of such a definition are considered, it cannot appear strange that this should be the case. In the first place, the language which is usually employed is either in itself so equivocal, or rendered so by abuse, that misconceptions and mistakes can scarcely fail to occur. In the second place, the ignorance of most medical men on the nature and construction of mind is so general and so great, that few of them can either appreciate, or employ strictly philosophical language when on the subject of mind. And, lastly, the properties of mind are so peculiar, so diversified and so opposite to those of mere matter, with the laws and qualities of which almost all our ideas are intimately connected, and from our knowledge of which almost all our language is formed, that it is generally difficult, and sometimes impossible to employ such terms as sufficiently express even the imperfect ideas which we entertain of the laws and operations of mind. In proof of the first and last of these positions it may be observed, that melancholy, derangement, insanity, delirium, crazy, and the majority of such terms as are employed to signify a diseased or unnatural state of mind, are derived from different forms and states of matter, and are so loosely employed as to convey a very indistinct notion of the metaphysical conditions which they are intended to denote. Thus *melancholy* was adopted by the Greeks, from their belief that black bile was the cause of this disease. *Derangement* has been taken from the French, and merely signifies out of rank, or order. *Insanity* is derived from the Romans, and means simply unsoundness, without reference to any object. *Delirium* is from "de lira," out of the track, and owes its origin to the process of ploughing, in which the horses are made to travel in a certain line: and *crazy* is from "ecrasé," which is applied to any thing shattered or broken. As to the second point—that medical men are sadly defective in metaphysics—there is, we fear, but too much proof. The great mass of our profession study medicine just as the shopkeeper studies grocery, or as the artisan studies his trade, and man is regarded as a machine, differing in little from any ordinary piece of mechanism, beyond its superior delicacy or its more easy derangement. All his functions are regarded as physical processes; some of them refined and recondite it may be, but still referrible to mere material principles, because ostensibly executed by mere material agents. Chemistry can illustrate one class of phenomena, pneumatics can unfold another, while by hydraulics and mechanics, capillary attraction and elasticity we can arrive at the solution of as many more. The agency of mind, as an independent principle, is seldom thought of and more seldom known; and although, perhaps, the majority may be not only ready to admit, but anxious to maintain the compound character of man, the most active and the most influential half of him is often wholly neglected and very rarely studied, or understood. Hence is it that we are so perplexed, so confounded with unmeaning language in six-tenths of the volumes which issue from the medical press, and hence is it, more especially, that our views of insanity are so inconsistent and so incomplete. One gravely divides insanity into *ideal* and *notional*, thus giving to notion and idea, words as nearly synonymous as the English language can furnish, two very different and characteristic meanings. Another tells us that madness is false perception; a third that it is an incorrect association of familiar ideas; a fourth that it arises from intensity of idea; a fifth that it is drawing fair conclusions from false premises; and a sixth, believing that folly and madness are two very different things, teaches us their difference by saying, that a madman draws fair conclusions from false premises, while a fool draws false conclusions from fair premises. It requires some degree of fortitude to oppose the authority upon which the last definition rests; but we believe that the foundation of the distinction which has been thus drawn by Locke is fanciful. He, who draws a fair conclusion from false premises, has a defective perception; but he, who draws a false conclusion from fair premises has a defective judgment. The one is misled by the fallacious character of his perceptions of things; the other by the fallacious inferences which he makes from perceptions faithful to Nature. A man, consequently, is a fool who judges inaccurately, and a madman is insane because he perceives inaccurately. We shall afterwards, however, have an opportunity to shew that a fool is generally not less deficient in his perception than a madman, nor a madman less faulty in his judgment than a fool; that madness and folly are but *different* degrees of the *same* disorder. Beddoes might with great justice, therefore, observe that "mad" is one of those words which mean almost every thing and nothing.

"I cannot say that I obtained much help from the definitions given by different medical authorities; for not only were some of them, as Dr. Good has truly observed, 'so narrow as to set at liberty half the patients at Bethlem, or the Bicêtre, and others so loose and capacious as to give a strait-waistcoat to half the world;'* but I found that when medical men were required to explain what meaning they attached to the word Insanity, they generally satisfied themselves by giving such as had been repeated by one author after another, apparently without examination; and I observed, that the practical decisions to which they were consequently led, often involved them in inconveniences, of which some had become so apprehensive as to abstain, professedly, from venturing upon any definition at all; endeavouring to content themselves, and to close the subject to all other inquirers, by asserting it to be too mysterious for man to understand. Yet I could not divest myself of the impression that the subject was not understood, only because it was not made the subject of that kind of investigation by which medical men attained a knowledge of any other subject connected with their profession; and that if they would attend more to the true physiologists of the mind, the writers on mental philosophy, who had investigated the nature and order of the mental functions, and would also observe the manner in which their own minds were exercised, they would not find it more difficult to mark and to comprehend the departures from the healthy performance of these functions, than the deviations from healthy digestion or respiration." 294.

It will be admitted that he, who requires to describe and understand diseased functions of whatever organ, should first be well acquainted with their healthy state; yet this reasoning, when applied to derangements of the mind, is equally correct and still more forcible. If the natural and healthy structure of mind be unknown; if the nature and relationship of its various faculties be unattended to; if the origin, development and association of its ideas be unrevealed; if the extent and boundaries of its perfect action be untaken; and if it be more a subject of conjecture than of certainty, when its operations are disordered and its functions in an unhealthy state, how is it to be supposed that we can pronounce, with any satisfaction to ourselves or any safety to our patients, on either the existence, the degree, or the duration of their derangement. Dr. Conolly has, therefore, done a very important service to the profession, in calling their attention to the construction and properties of mind. By investigating its history, from its most perfect state, through all its modifications of strength, and through all its varieties of disease, until it becomes affected with confirmed madness, he has laid the ground-work of the only successful, because the only scientific mode of investigating insanity; and while he has pointed to the means of unfolding many of those mysterious arcana in the history of this malady, which have hitherto perplexed and mutilated every inquiry, he has opened up the way to a more rational, because a more metaphysical mode of treatment. The consequences which have flowed from ignorance on these points have been truly frightful. People, as safe and as sound as any of their neighbours, have been pronounced deranged, have been torn from their homes and families, and have been entombed alive within some solitary receptacle of insanity, there to consume a wretched existence in darkness and despair, frequently unpitied and not unfrequently forgotten; while others have been permitted to stalk abroad a nuisance to society and a burthen to themselves. And surely of all forms of entombment, none can be so frightful as that in which both mind and body are interred together, and in which the unfortunate and unheeded victim goes down to his living

* Study of Medicine, vol. IV.

grave conscious of his sanity, yet confirming by every fresh argument and remonstrance the doleful judgment, which ignorance, or something worse than ignorance, has pronounced against him. The funeral rites of the deluded Indians are humane on the comparison. At such triumphs of superstition life is voluntarily resigned because considered forfeited, and the surviving partner descends with the fortitude of devoted feeling into the same cemetery with the deceased. But here there is neither love, nor religion, the enthusiasm of venerated prejudice, the force of patriotism, nor the law of custom, to sanction or to palliate such an act of cruelty. National feeling and individual safety, it is true, are pleaded in excuse, and the ostensible motive for taking a step so decisive, as that of enclosing a human being for life within the walls of an asylum, is the desire of preventing the diseased from either doing injury to himself or others. But we would ask these humane life-guards of the species, what class of the insane generally occupy the cells of lunatic asylums? Are they the violently deranged? Are they such as are calculated to infuse terror or excite suspicion? On the contrary, are not the great majority of them as peaceful and as harmless as any members of society? Without cunning to devise, or power to execute any dark deed of mischief, they are often neither able nor disposed to injure, or alarm. Labouring under a kind of second childhood, many of them are much more innocently inclined than thousands going at large, without the precaution of a strait-waistcoat, or the luxury of a shaved scalp. The dangerously deranged form but a small complement of this idiotic population—not more than perhaps two-tenths—yet, according to the present system, all these differently disposed and variously affected lunatics are crowded together into the same apartments, indiscriminately exposed to the same society, and to a very great degree are treated in the same manner!

Dr. Conolly defines insanity to be "the impairment of any one or more of the faculties of the mind, accompanied with, or inducing a defect in the comparing faculty." If this definition be admitted it will involve in the charge of insanity many who think themselves paragons of wisdom, and will reduce the number of our σοφοι to a very lamentable fraction of the general population. Every man, who cannot compare one simple idea with another simple idea, so as to draw a true and natural conclusion from them, is insane. Every vain and impertinent coxcomb, who so overvalues his own pretensions, or so undervalues those of others, as to infer his superiority; every gay and harmless belle, who can see no beauty but her own; every uxorious domesticated peasant, who considers his wife as the prettiest woman in the world; and every fond affectionate father, who regards his children as examples of amiability and patterns of talent—all these, et hoc genus omne, this sweeping definition will stigmatise as possessing the elements of ordinary insanity, and as labouring under the same *kind* of defect in their comparing faculty, which constitutes madness! If a defect in the power of forming just comparisons be really the essence of insanity, the truly sane will be cast woefully into the minority, as we pledge ourselves to discover more madmen in silk than in strait-waistcoats, more wrongheaded people at large and unrestrained, than immured in cells and jacketted in darkness.

The spaciousness of this definition will, probably, on all these accounts, be objected to. Every man naturally likes to limit the sphere of madness as much as possible, and however he may be induced to consider others as "a little touched," he cannot easily be prevailed upon to think similarly of himself. Yet the principle and spirit of this definition appear well founded, and we do think that the professor has been led to its adoption from an extensive analysis of human life. In every instance of insanity which occurs to us, there has been some defect in the power of making fair comparisons; yet in strict parlance it cannot be said, that every defect in the comparing faculty constitutes insanity. To this length it is not likely the Dr. intended to go. It is the *extent* to which this defect exists which determines the person to be insane, or otherwise. A man may have a very weak and imperfect power of comparing, he may draw many false comparisons, and entertain many erroneous views; yet he may be very far removed from insanity. It is only when this power is so shattered and impaired, that he systematically misjudges upon some points, or so generally misconceives all, that no degree of public faith can be reposed in what he says, nor any confidence of security in what he does, that he can be esteemed insane, and no longer capable of occupying a place in rational society.

On points of so much abstruseness as the present, definition-making is dangerous work, and we dislike it; believing that it is quite possible both to understand and describe a point or thing tolerably clearly, without being able to define it; and convinced that, while a good definition may prevent the trouble of a tedious description, a bad one can neither serve for description, nor definition, and is only calculated to mislead. When we are told that insanity is the impairment of any one or more of the faculties of the mind, accompanied with, or inducing a defect in the comparing faculty, the phraseology is very little more than intelligible. In the first place, to think and speak metaphysically, and of course on a point of this nature, no other language than what is strictly metaphysical should be employed, there is no such primary mental power as a *comparing faculty*. The only faculty of mind, which can compare and infer resemblances or differences, is *judgment*; and every act of comparison which we make is performed by judgment. Secondly, supposing that we had a comparing faculty, what is to be understood when it is said, that the impairment of *any one* or more of the faculties of the mind, inducing a defect in the comparing faculty—*another* supposed faculty of mind—constitutes madness? Might it not be said with as much consistency, that the absence of *any one* of all the letters in the alphabet, causing the absence of *another* letter in the alphabet not included in any one of all the letters of the alphabet before-mentioned, constitutes an imperfect alphabet? The comparing faculty is necessarily included by the phrase "any one," otherwise we might say "a whole plus a part," which mathematicians would probably suspect to be but indifferent algebra! And, in the third place, we believe that insanity may occur without the derangement of any other faculty than that of judgment. Whatever *other* faculty may be affected in madness to a certain extent *judgment always* is, and hence the main principle of Dr. Conolly's definition is correct, however we may gainsay its phraseology; but we may have any one of all the other faculties affected without the judgment being seriously implicated. The man, who is defective from age in his perception, or memory, or association, may be perfectly rational. It is true, he cannot draw any comparisons between objects which he cannot accurately perceive; but that is not a positive, it is a mere negative defect, and, unless the man so circumstanced should insist, either that his perceptions were perfectly correct, or that he was perfectly qualified to draw inferences from them, the mere failing in his faculty of perception would not constitute him a madman. In thus criticising the Dr.'s definition we mean neither severity, nor censure. The disease, which he undertook to define, has baffled many talented metaphysicians; and failure must now be less acutely felt, since it has been so frequently experienced. Believing that insanity is only the superlative of minor degrees of mental weakness, and that some of the elements which enter into its composition may be discovered in the most of minds, we despair of ever meeting a definition of this disease, which shall include every case, and to which no exception can be taken.

"One man suffers an impairment of sensation, sees what has no existence, or hears sounds which are unreal; and he believes in the reality of his visions, and of the sounds which come to his ears. He has, then, an impairment of *one* faculty, accompanied with a defect in his comparing powers on the subject which that impairment affects; and on that subject he is not in his sound mind. His memory, except of the false impressions, his imagination, except as regards his delusions of sense, are not affected; and in all subjects except that, he is a reasonable and sane man. Another man appears not to see what is present, or not to hear what is said to him, or not to know where or with whom he is, or how he came to be where he is, or wherefore; he has no thought of his relations, his friends, the occupations of his former life; he imagines himself a great general, or an emperor, or possessed of boundless wealth, or power; yet he is poorly dressed, his hands are confined, he is controlled by keepers, separated from all his family, and in all things guarded and watched as a prisoner, or as a child. Here we have an affection of all the faculties of the mind, that of comparison of course included; and this man is unable to exercise any of his faculties, or that comparison, on any subject; and is therefore insane on all. Between this extreme case, and the slightest case, there may be many varieties of insanity;—on one subject; on two subjects; on all the subjects of sense, and not on those placed in the memory before that sensorial delusion existed, or, without delusion of sense, an excited imagination; in fact, any affection of any one or more faculties, which is accompanied by, or induces, a defect in the comparing powers." 302.

But the man with impaired sensation, who attempts to compare two objects which are subjected to his senses for examination, must inevitably make a false comparison, and the defect in his judgment may be limited by the degree of impaired sensation under which he labors. The lunatic, who neither sees, nor hears, nor perceives, nor recollects any thing distinctly, but fancies things which never existed, cannot compare properly; for he has no legitimate data for a comparison. The man, who believes his legs are made of butter, who wraps them up carefully, and surrounds them with a wooden box, cannot judge correctly; because his morbid sensations, or his diseased imagination, apart from sensation altogether, have made him to believe that his legs are not like the legs of any other person. The data whereon he is to ground his comparison are false, and there is no necessity that the comparing faculty, even supposing that there was such a faculty, should be radically defective, to account for the deficiency of the comparison. The judgment is misled by the deceptiveness of the perception, or the ardour of the imagination, and the comparison which it makes is necessarily false; but beyond this sympathizing error, there may be no radical defect in the construction of this faculty. In some cases we find one faculty diseased, in other cases several, and in some all; but whether the affected portion of mind extend to all, or be limited to one faculty, the judgment, apart from any idiopathic derangement, will be affected precisely in proportion to the impairment of the other faculties, and just in virtue of that impairment. One of Napoleon's generals thought he heard the people salute him as their king. The general's sense of hearing and, probably, his imagination were deranged, so that his judgment could not compare the evidence which his hearing furnished with that of his sense of sight, which otherwise would have rectified the fallacy. Mr. Bayle mentions the case of a lady, who put pieces of flint into her drink, believing them to be lumps of sugar. A Prince of Bourbon, believing himself to be dead, refused sustenance, and to prevent his dying of starvation two persons were introduced to him as inhabitants of the world of spirits, who invited him to dine with another of the illustrious dead—Marshal Turenne. By this artifice the Prince was preserved alive, and until this delusion was dissipated, it was found necessary to invite him daily to meet at dinner some ghost of reputation. Yet, says D'Israeli, in the ordinary business of life, the Prince was sufficiently collected to transact his own affairs. It is very difficult to say what faculty was in this instance primarily deranged, but the power of comparison was on this point evidently lost, rendering it impossible for him to discover that a man, who ate, talked, walked and engaged in business, could not possibly be a subject of the realm of Pluto. A case precisely similar to the Professor's is given us by *Lemnius;* and the following still more curious instance of the same delusion may be found in *Heywood*, on the *History of Angels.* A young man believing that he was dead, abstained from food and importuned his parents that he might be carried to his grave and buried, before his body became offensive. Following the advice of his physicians he was accordingly laid upon a bier, and was carried towards the church; but the procession was met by two or three merry fellows employed for the purpose, who asked the name of the deceased; and, being told by the bearers, replied—"Well, the world is happily rid of him, for he was a man of a wicked life, and his friends have cause to rejoice that he did not make his exit at the gallows." The supposed corpse hearing this attack immediately raised himself upon the bier, and said that "he had never deserved the character they gave him, and that if he was alive, as he was not, he would teach them to speak better of the dead;" but, the men continuing to abuse him in the same opprobrious language, he could bear it no longer, and leaping down from the bier, fell upon them with great fury, and beat them till he was quite tired and convinced that he was as actively alive as he ever had been. A young clergyman, when about to be married, received the charge of a gun in his forehead. After some days of dangerous illness he recovered his health, but was deprived of his understanding. From the period of his recovery until his eightieth year, when he died, the prospect of marriage never left his sight; he talked of nothing else, and in the midst of age and decrepitude he fancied himself a young and active bridegroom. Dr. Conolly's theory of this hallucination is ingenious.

"The wound had in this case produced some change in the nervous organization, preventing, from the time of its reception, the accession of any new idea: nothing was ever more received which could be compared with the past, nothing added to the stores of memory to be compared with former accumulations; consequently, nothing to mark the flight of time: and nothing was perceived of all those circumstances, which, if seen and compared with the single impression seemingly remembered, would have shown that since it was made years had passed away, and had wrought their usual changes." 329.

Another clergyman, on thoughtlessly swallowing the seal of a letter which he received while at table, became so frightened by his companions, who told him that it would certainly seal up his bowels, that he ever after refused food and literally starved himself. As his medical friends had administered purgatives during his illness, hoping to convince him that his fears were groundless, had this man been able to compare the nature of his supposed complaint with the character of the remedies employed for its removal, he would have been convinced that, had his bowels been so artificially confined, purgatives would have found great difficulty in opening them, or if they should have succeeded, their success would have been a proof that he might have taken food with impunity. Dr. Burrows mentions the case of a lady who, imagining that a tooth, which she had extracted, had fallen back into her throat, insisted that she was no longer able to swallow, although she continued to eat and drink as usual. One man believes himself to be a tea-pot; another a barrel; a third thinks he has a cobbler working in his stomach; a fourth is convinced that his hips are made of glass; a fifth believes himself to be a king; a sixth thinks that he is God, or the Son of God and sovereign of all things. All these have evidently lost the power of comparison on the several subjects of their derangement, and this loss of the power of comparison arises from the impairment of one or more of the mental faculties, more especially judgment.

The author seems anxious to expose the fallacy of the general impression, that "poets are half mad," and that to become a poet the mind must be somewhat cracked. On this point we are not quite certain whether we comprehend the Doctor. If he mean to assert, and we think he does, that the first rate poet—the man of a century—is not cracked, we agree with him. But if his opinion be that all whom the world call poets, using the ordinary signification of that term, are not only perfectly sane but men of the soundest judgments, we are decidedly of another mind. Milton and Shakespear, Homer and Virgil, and perhaps one or two other favored souls of heaven whom we are afraid to guess at, were writers as remarkable for strength of judgment and accuracy of perception, as for the brilliancy and force of their imagination. They were, therefore, neither eccentric, nor insane. Every mental faculty was duly proportioned, and every mental faculty was in the superlative degree. The ardency of their fancy did not mislead their judgment, the brightness of their imagery did not seduce their taste, and the worlds which they created had nothing in them, which was not perfectly consistent with every object of sense. But can all this be said of the whole herd of poets? Can it be said of Dryden or of Savage, of Collins or of Cowper, of Swift or of Goldsmith, of Alfieri or Petrarch? Can it be said of Byron, whose poetic wing rose as rapidly, soared as high, and tracked its progress with as many victories, as ever distinguished any favourite of the Muses? In short, can it be said of any save themselves? The fact is that, with a few and a very few exceptions, the popular sentiment is an expression of the general rule, and its accuracy is too amply attested by the history of poetry. A defective judgment, or, what amounts to the same thing, an inordinate imagination is visible in every department of the poet's history. A contempt of foresight, a want of prudence, a recklessness of consequences, a thirst for pleasure, an improvidence for the future, a forgetfulness of the past, and a love for the present, constitute the leading features in a poet's portrait. And whence arise all these properties? From a deficiency in the judgment, a preponderancy in the imagination; and let this deficiency of the one and preponderancy of the other be increased, and often but a very little, and poets and poetry may be easily converted into idiots and nonsense. In what were Laura and Stella superior to the Dulcinea del Tobosa of Don Quixote, and in what do the inspirations of Alfieri and Sacchini differ, but in degree, from the convulsive ravings of the ancient priestess at Delphi or Dodona?

Cases not unfrequently occur in which the judgment is so far injured as to make false inferences from very intelligible and simple premises, but in which an obvious struggle may be observed in the reasoning faculty to regain its influence. A man, considering himself insolvent, became deranged, and for some time laboured under the impression that he was unable to meet his creditors. By having his debtor and creditor accounts, however, placed before him, and by satisfying him that the balance was evidently in his favour, he was recovered from the delusion, and restored to his ordinary state of mind. Pascal was occasionally annoyed by the fancy, that he stood on the brink of a frightful precipice. The delusion was removed by his servants placing a chair on the supposed site of the dreaded danger.

When a practitioner is called to attend a person supposed to be deranged he has two points to engage his attention—first, to ascertain whether the individual in question be really of unsound mind; and, secondly, to decide upon the nature of the treatment adapted to his case.

"If what has been said concerning the mind in health, and concerning its inequalities, weaknesses, and peculiarities, and unsound state, contains correct views of the subject, I do not think that in any case much doubt can long exist. The patient should by all means have fair play: there should be no trick, no delusion, no artful excitement or provocation, no deception of any kind put upon him. Pains are often taken, by those who are anxious for the removal of the individual, to influence the mode of introduction of the practitioner, and the way in which he speaks to his patient; and there is no occasion on which he is more called upon, by his duty, to exercise his authority." 365.

"The fact of the patient's madness can only be established by certain tests of the manner in which his intellectual faculties are exercised, and these tests are to be found in his appearance, in his dress, in the known physical accompaniments of madness, and in his words and actions. *That* is the medical question. The *next* is a medico-legal question, and turns wholly on the disposition of the patient to injure himself or his property, or to injure others and their property; and on the probability of such a disposition, though not manifested, being suddenly developed. On the first question hangs the medical treatment and superintendence; on the second, restraint, confinement, deprivation of authority, and control over property. Medical care and superintendence may be necessary in every case; but the mistake has been to conclude, that restraint and the other circumstances are also necessary, which they certainly are not." 373.

"Let me repeat, that the medical man has a plain duty to perform, which requires no arts of this kind. He has to ascertain whether or not the functions of the intellect are disturbed, and require the aid of medicines directed to its relief by certain effects they produce on the body: and he has to determine whether the degree or character of the disturbance is such as to make the patient dangerous to himself or to others, either as regards person or property. The decision of the first question is often quite distinct from that of the second: the interference of medicine is required in most cases of disturbed mind; personal restraint may be required in many; but the degrees of it which are required in different cases vary, as the cases themselves vary, from the slightest to the most complete; and complete restraint is very rarely required." 386.

The medical treatment should vary with the cause of disease and the nature of symptoms. One man is melancholy and morose, another is mirthful and jocose; one dwells with unwearied pertinacity upon some favorite topic, while another runs from point to point with exhaustless caprice. This man is so wild as to require restraint, that so diffident as to need encouragement. The entire mind is unhinged in one case, a single faculty is disordered in another. Is it, therefore, to be conceived that, where the manifestations and degrees of disease are so multifarious and so different, the same plan of treatment can be generally beneficial. Sometimes insanity arises from misfortune, and sometimes from prosperity; often from mental and frequently from physical causes; now it is chronic, again it is acute; in this instance it appears in an old and debilitated, in that in a young and active constitution; now it assumes a form mild and moderate, then all is violence and and fury.

" When an unruly patient enters a common lunatic house, he is bled, dressed in a strait-waistcoat, has his head shaved, is subjected to the shower-bath, put upon low diet, kept in darkness, and compelled to swallow some active purgative medicine. If measures of this kind, which may be well enough suited to active delirium, do not effect any amendment, the medical resources of the establishment are at an end. Starvation, imprisonment, loneliness, and threats are then resorted to; or if the proprietor of the house happens to be very alert, some desperate, or some unjustifiable experiment is tried; whirling round upon a horizontal wheel, intoxication, or some strange method of astonishing the patient; such as leading him blindfold and headlong into a cold bath. At last peace is effected. The patient is exhausted; or his excitement is succeeded by what is called the low state; or he has learned cunning, and moderates his actions. In a few cases, the disease is soon at an end, and it is possible the amendment may be perceived, and the patient restored to his family: *possible*, but not as a general fact, *probable;* for the patient is seldom seen by those who are judges of his amendment: a few minutes every two or three days seeming to be the maximum of medical attendance in the best circumstances; and many weeks, or months, passing over in other cases, without the patient being seen by any medical man at all. Too often, the low state, considered but a continuance of the malady in another form, is succeeded by another paroxysm of excitement, and the rest of a miserable life is passed in hopeless alternations between the two." 16.

This is sad and melancholy management, and, if it do not often make more madmen than it cures, the minds of those, who are usually sent for safety to an asylum, are less insane and less easily deranged than such a measure should require. What sad routine doctoring is it to shave and bleed and imprison every unfortunate wretch, whom severe treatment may have worried into violence! The custom of subjecting all kinds and degrees of insanity to darkness, seclusion and restraint, must either have originated from the conviction, that insanity is a hopeless incurable malady, or that treatment has such trifling influence over it, that it is of little moment what plan should be employed. The man who is idiotic and harmless can never be much improved by confinement, and neither the safety of those around him nor his own requires it. If misery, misfortune and distress have unhinged the mind, or if continued melancholy have undermined the judgment, is it rational to suppose, that a continuation of the very same causes which have induced such states should promise any thing but increased disorder? Few, we believe, have judgments so consolidated, that unfavourable circumstances may not weaken them. If this be true with respect to minds perfectly untouched, how much stronger is the observation when applied to minds already seriously impaired? What is there in an ill-conducted lunatic asylum at all calculated to recover the wandering mind? Is an undisciplined reservoir of madmen the place which should be selected for its abode; is a noisy and distracted companionship the society which should be chosen to entertain it; is solitary confinement the treatment which should be adopted for its cure? If, indeed, the case be confirmed and supposed incurable, then every plan of management may be equally availing; but if the faintest prospect of returning reason be entertained—and we ask when should it be entirely abandoned—an ill-regulated lunatic asylum is of all places for the insane beyond all comparison the worst. The Greeks sent their insane to the island of Anticyra, which was remarkable for its crops of hemlock; and there, removed from every cause of irritation and in the free enjoyment of rural liberty, many, it is reported, were restored to health.

——— Verum ambitiosus et audax
Naviget Anticyram.

But we have no Anticyra for our patients. We bury them in cells, exclude them from all rational companionship, crowd them together, and doubly curse them with society as irrational as their own, and we call madness a hopeless mischief which no art can cure! The dogs, which crowd our streets in a rabid state, are treated with more forbearance. They are suffered to mix with rational society; and, while a father or a husband is doomed to the dungeon and the jacket, the lapdog is kept at home and permitted to enjoy the attentions of his mistress. Except the transient voice of a passing visitor, or the authoritative accents of the keeper, these wretched outcasts from society hear little from day to day but the unconnected ravings of disordered intellects, nor do they see any thing but the grotesque and gloomy figures of the possessed. Not a word of kind encouragement, not a sentence of rational speech, not a look of condolence, nor a sigh of pity, is heard or seen. All within them is waste and vacuity and disorder, all around them is as irrational as themselves, and the errant mind is permitted to deviate, untutored and unchecked, into all the bewildering labyrinths of a diseased and capricious fancy. It may be wise to crowd our fevers into the same pesthouse, and to trammel the power of contagion by cabelling it out at sea; but it is in open violation of the first principles of metaphysics, to associate every variety of mental derangement and eccentricity. Patients labouring under the same fever can communicate no injury to each other. They are all similarly diseased and similarly circumstanced, and however they might prove injurious to others, they cannot be hurtful to themselves.

But, in every instance in which mind is affected the case is different. The principal of imitation is exceedingly strong in the breasts of most lunatics; the peculiarities of one are aped by another, the contagion of example spreads from cell to cell, and where there is not a ray of reason to guide the wandering intellect from its favourite estrangement, every extravagancy is made the subject of imitation, and faculties, which were at first only disordered, become ultimately diseased. Some of these abuses are forcibly and eloquently exposed by the author; but we were somewhat disappointed on finding so small a portion of his work devoted to what appears to us the most interesting, because the most practical department of his subject. In 33 pages he discusses points which might well have occupied three times that space, and although the topics which follow lay claim to serious consideration, yet the reader can be more easily convinced of the necessity of reform and of the degree to which it should be carried, when the nature and extent of existing evils have been fully pointed out.

Even in those cases in which restraint may be rendered necessary by the violence of the symptoms, a mild and soothing manner, aided by proper medicines, will often produce a much happier effect, than constant darkness and severe discipline. If restraint be continued after the violence of the paroxysm has subsided, and the patient becomes enabled to perceive the ordeal to which he has been doomed, irritation is improperly added to distress. The very important and practical fact that insanity, even in its worst form, is nothing but the superlative of lower and less intense degrees of derangement, should never be absent from the mind of the practitioner. If properly attended to it will inform him, that lunatics are not those anomalous phenomena in the records of disease, not those fallen stars from the sphere of reason, which they have been too often wont to be considered—that they are only a few steps removed from the most sane and talented among us—that no lunatic, however violent his symptoms, however shattered his judgment, however malicious his propensities, or however reckless his passions, should be thrust forth from the world as a deadly leper, no longer tolerable in the haunts of men, as an irrecoverable alien, who has lost his social badge, and can never more be restored to the bosom of society. It will teach him to adopt every art which may banish the distracting hallucination from the mind, which may heal irritated feeling, sooth intemperate violence, encourage despondency, and bring back to soundness and to health the wandering intellect. Above all things it will convince him that restraint and confinement are among the last and least hopeful resources to which he ought to look for aid; that they are only useful by preventing danger, never by removing the disease. Unlike almost every other remedy against disease, the dungeon and the jacket are never more than negatively good. Tonics may remove debility, bleeding may cure inflammation, but the cell and the waistcoat can never affect more, even when judiciously applied, than preserve from absolute destruction. Whenever this is apprehended it is wise and well to have recourse to them; but as soon as the danger has passed away, and the moment of its removal should be carefully watched, such violent measures should be succeeded by milder treatment. It is only *after* their employment that a cure can be attempted. While they are required curative treatment is at a stand, and it is itself no very harmless exhibition of derangement, though perhaps not of lunacy, to expect to cure madness while jacketted in a dungeon.

"Discouragement is often thrown on *mental* treatment by injudicious attempts to act on the faculties of the mind in these cases of weakened organization. Such attempts cannot be made with success before the bodily state is improved, and, if the bodily state cannot be improved, such attempts can never succeed at all. The power must be in some degree restored before we can reasonably call for action. When some degree of power *is* restored, by good air, careful diet, tonic medicines, the shower-bath, sea-bathing, and attention to every article of regimen, then we may begin to exercise the faculties a little; but this must be done with the most extreme caution and tenderness. It is quite in vain to attempt to do it on any rigid and formal system. It may be practicable to-day, and impracticable to-morrow. Times, and opportunities, and occasions of returning strength, must be watched for, and profited by, but not too zealously or vehemently. To establish this system of treatment and watching is the duty of the practitioner; and he *may* establish it, if he does not send off the patient to a place in which such a system cannot be pursued. Whether it is immediately practicable to endeavour to rouse the mind, or some preliminary attention is required before such an endeavour is practicable, or whether, as will very often be the case, it is desirable to abstain from direct attempts to influence the mind, and necessary to divert it, in its weakened state, like the mind of a child, to objects unconnected with the morbid ideas;—in all or any of these cases, the patient requires that degree of care, and watching, and that medical and mental management which, difficult in any circumstances, are impossible in a house full of lunatics." 398.

By making a timely and judicious appeal to the mind Cowper was recovered from one of his most inveterate fits of despondency. A lady, who believed she saw the ghost of her husband, who was still alive, was recovered from her delusion by the husband appearing before her and entering into conversation about the affairs of their family. A man, who lived opposite the stall of a cobbler, and was accustomed to look out at him while at work, became impressed with the idea that the cobbler, in consequence of having absented himself from the stall for a few days, had been actually swallowed by him; but on giving him an emetic, and bringing the cobbler suddenly before him, as though he had been thrown up during the action of his medicine, he got better and his delusion left him. Such cases of partial derangement and of complete recovery are endless, and surely it is sad and melancholy discipline, which would restrain such monomaniacs, who are as unoffending to others as they are harmless to themselves. If a man be lunatic once a month, or once a week, or even once a day, is that an argument which should be sufficient to induce us to doom him to uninterrupted misery? The very same reason, which calls upon us to confine him during the fit, calls upon us to liberate him after it; and it is an inexcusable abandonment of all feeling to stigmatise a fellow creature with the frightful charge of madness, and to treat him systematically as insane, when, except at specific intervals, or on specific subjects, he is as sensible, if not more so than many of his neighbours who esteem him mad. Such random, routine management is sanctioned in France; but it is too gross to be tolerated in England, were its extent generally known, or its consequences properly understood. We fear that the greater part of the following passage, which appeared in the London newspapers some years ago, is still applicable.—"Notwithstanding the recent regulations there are many private madhouses in the neighbourhood of the metropolis, which demand a very serious inquiry. The masters of these receptacles of misery, on the days that they expect their visitors, get their sane patients out of the way; or if that cannot be done, give them large doses of stupifying liquor, or narcotic draughts, that drown their faculties, and render them incapable of giving a coherent answer. A very strict eye should be kept on these *gaolers of the mind;* for if they do not find a patient mad, their oppressive tyranny soon makes them so—there should be no such receptacle as a private madhouse allowed; and the relations and friends of the insane should be allowed to visit at all times." We do look forward with anxiety for the day, when lunatic asylums shall be managed with the same humanity, science and good sense, which have distinguished and raised the other public charities of this country above those of every other nation.

As the author very properly observes, justice and humanity are the two principles which should ever direct our treatment of lunatics. Justice will prevent us from carrying our humanity to an extent, which might endanger the safety either of property, or life; and humanity should induce us to do every thing for the comfort, as well as for the cure of the deranged, which is not incompatible with either of these objects. Enough, we presume, has been said to show that the present system of treating the insane is woefully deficient in both these points, and if the objections which have been urged against it have been well grounded, the mode in which some of them may be obviated it will not be difficult to lay down. If it be an error to entrust into the hands of non-professional men the charge of insane people —if it be an evil to associate lunatics with lunatics, without any respect to their different forms, or degrees of lunacy—if it be a defect to have lunatic houses in the uncontrolled possession of interested men—and if it be true that the present system of seclusion prevents even professional men from studying with proper attention the nature and cure of lunacy—surely these are evils well worth the people of England to investigate and to rectify, as well from the sad extent to which they are carried, as from the disastrous consequences to which they tend.

The substance of the arrangements which the Professor adopts to effect all these objects is—to place all insane persons under the care of the state —to have a lunatic asylum in each county, two in London, and one, if necessary, in any large provincial town—to have small lunatic houses in the neighbourhood of each of these asylums, which should be under the same government with the larger houses, and into which such patients might be admitted as require removal from home, but whose friends are averse from sending them to the asylum—to make every lunatic asylum the property of the state—to send no lunatic to an asylum, except it were found from his relatives and friends that he could not receive proper care elsewhere—to make every lunatic asylum a school for the knowledge of lunacy—to have all the officers of these asylums appointed by, and under the control of the Secretary of State—to attach to every asylum a certain number of medical officers and keepers, who should be ready at all times to attend insane patients in their own houses—to give notice at the public asylum of the district, as soon as signs of insanity appear in any person, and, if a keeper be required, to have a certificate to that effect signed by the medical attendant on the patient's family, and some of the medical officers of the asylum—to keep a register of every insane person, whether in or out of the asylum, and to have the out-patients visited by a medical officer from the asylum, at least twice a month in chronic, and once a week in recent cases—to have non-professional men as visitors attached to each asylum, who should visit the patients in the asylum once a week, those out of it twice a month, and make a monthly report of the state of each—to prohibit the reception of lunatics into work-houses, or houses kept by persons interested in keeping lunatics, and to punish all instances of concealment—to so separate the insane, that no society could be held between the deranged—to allow each lunatic a keeper, who should be with him a great part of each day, to converse with, sooth, amuse, and instruct him—to open every asylum to the public from two till four, p. m., three days in the week, a keeper or house-pupil going round with the visitors—to allow free access to the friends of the insane—to have divine service regularly performed in each asylum—to lay before the Secretary of State every quarter a report of the number of patients admitted and discharged from each asylum, with the names of the out-patients and visitors.

How far these suggestions might tend to obviate the evils which have been pointed out, the reader may now be tolerably well prepared to form his own conclusion. It appears to us that, although strong exceptions may be taken against many of them, their general principle is excellent. The preposterous custom of delivering the insane into the hands of men, whose utter ignorance of mind, as well as of bodily disease, disqualifies them from taking charge of patients, who require both medical and metaphysical treatment, would thus be abandoned, and interest would be no longer allowed to traffic with liberty and life—professional men would be furnished with the means of studying every form and modification of insanity, and of preparing themselves for meeting and managing it in private life—the peculiarities, tastes, habits and propensities of individual lunatics would be carefully consulted, and every light which they might cast upon the nature of their derangement could be made available—friends would confide with comfort and satisfaction in the integrity of those to whose care they had committed the insane, and the insane would look with confidence and affection on their attendants, as men uninfluenced by any other motive than that of discharging with humanity and success the important duties which devolved upon them. If any thing can solace the mind of a lunatic during the lucid intervals with which he is occasionally blessed, and can reconcile him to the sad bereavements which he is doomed to suffer, it is his conviction that, when his lights have been extinguished and he lies helmless and hopeless upon the surge, there is a skilful hand prepared to rescue him from the tempest, or, if unable to save him, anxious to assuage his misery and to sooth his mind. And when it is remembered that the most furious insanity differs only in degree from passions, emotions and propensities, which the wisest among us are hourly betraying, and that the transition between a harmless oddity and a pernicious hallucination is as short as it is easy, those, who are blessed with the richest gift of heaven, should look with any other feeling than one of ridicule or indifference upon the distracted lunatic, and should hail with encouragement every plan which furnishes the faintest hope of ameliorating his condition, and of restoring him to reason. Dr. Conolly has espoused the interests of this most destitute class of the human race, in a style and with a

X 2

talent which are equally creditable, and we do earnestly beseech every friend of humanity and every advocate of improvement, to weigh the alterations he suggests, to consider the errors which he exposes, and to add their influence to his efforts for the relief of those, who of all patients are most unqualified to relieve themselves.

The language and style of this work are superior—very superior to those of any modern medical publication with which we are acquainted. Several of the propositions are, we think, Utopian—and, on the whole, the materials are much inferior to the workmanship.

